# Understanding COVID-19 vaccine hesitancy in health care professionals in Central and West Asia: lessons for future emergency mass vaccination campaigns

**DOI:** 10.3389/fpubh.2023.1196289

**Published:** 2023-06-16

**Authors:** Shalkar Adambekov, Alexander Bongers, Jonathan Hare, Dragoslav Popovic, Harsha Rajashekharaiah, Stefan M. Lawson, Giovanna Riggall, Larissa Kokareva, Brian Chin

**Affiliations:** ^1^Crown Agents, London, United Kingdom; ^2^Department of Epidemiology, Biostatistics, and Evidence Based Medicine, Al-Farabi Kazakh National University, Almaty, Kazakhstan; ^3^Asian Development Bank, Manila, Philippines

**Keywords:** vaccine hesitancy, LMIC, snowball sampling, communications, knowledge attitudes

## Abstract

A Quick Assessment of Vaccine Hesitancy approach was developed to collect population insights on vaccination hesitancy for low resource environments. Insights into COVID-19 vaccine hesitancy were collected through online webinars with heads of healthcare departments and anonymized online surveys of healthcare managers (HCM) and primary healthcare workers (HCW) in four countries in Central and West Asia (Armenia, Georgia, Tajikistan, and Kyrgyzstan) between 28 February 2022 and 29 March 2022. From the responses to the survey some key themes identified that underpinned in vaccine hesitancy across the region were perceived understanding of vaccine efficacy, conflict with individual religious beliefs, concerns for side effects, and the relatively rapid development of the vaccine and that improving communications strategies to address these concerns would be critical in combatting vaccine hesitancy through any future public health emergencies.

## 1. Introduction

Up until March 2023, 13.23 billion doses have been administered globally with 5.52 billion people, equivalent to 69% of global population having received at least one dose of a COVID-19 vaccine (1). Although significant progress has been made in upscaling COVID-19 vaccination globally, coverage in many Low- and Middle-Income countries (LMICs) lags behind developed nations, with 23.3% of people in low-income countries having received their full vaccination regimen (2, 3). Significant barriers to vaccination still exist in low- and middle-income countries and addressing them in detail is essential in reaching country immunization goals as well as WHO vaccination targets of 70% of the total population of every country while achieving 100% coverage among at-risk populations such as those over age 60 and healthcare workers (4–6).

Many barriers to increasing COVID-19 vaccination coverage can be broadly categorized into three main areas: (1) Procurement and supply chain, (2) Distribution, including cold chain management and (3) Socio-economic challenges including vaccine hesitancy. The challenges around procurement and distribution have been well documented (7) whereas capturing the intricacies of the socio-economic challenges is more complex and varies greatly across countries and regions.

Any response to vaccine hesitancy requires strategies that address the immunization policy, population-specific communication strategies, capacity building, behavior change, and collaborations across a wide range of stakeholders including health care workers (HCWs). Some of the considerations that can contribute to different vaccine hesitancy profiles include the accelerated timescale for development of vaccines, the utilization of novel technologies in vaccine development, structural and underlying lack of governmental or institutional trust in public health management, the interdependency with other preventative public health measures, and perceived (8–12).

Health care workers (HCWs) are at the forefront of nay healthcare emergency and yet are frequently expected to implement mass vaccination programs often supported with limited training, supervision, or guidance, as was observed with the COVID-19 vaccination programs (13, 14) coupled to HCWs being a priority group for receiving emergency use vaccines. This combination frequently leads to a situation whereby despite a high vaccination rate of HCWs this does not translate to high coverage among the general population and collecting insights on vaccination issues from HCWs could be important to inform policy on strategies to combat vaccine hesitancy in the general population (15).

In this perspective, the Vaccine Advisory Firm for Central and West Asia, a consortium of Crown Agents and FHI360, under a technical assistance project funded by the Asian Development Bank, describe the implementation of a Quick Assessment of Vaccine Hesitancy (QAVH) approach to facilitate rapid collection of population insights in to vaccination hesitancy and provides a summary of the responses received within across 4 countries in Central and West Asia (Armenia, Georgia, Kyrgyzstan, and Tajikistan) targeting key HCWs. Finally we propose that implementing similar cloud-based data input approaches targeting key populations can be used to provide a contemporaneous, cross-sectional snap-shot of opinions to augment traditional large knowledge, attitude, and practice (KAP) surveys when looking to formulate procedures for acute health emergencies.

## 2. Quick assessment of vaccine hesitancy

While vaccine hesitancy to routine immunization programs is well researched (16–18) the traditional profile of hesitancy does not necessarily directly transfer to emergency use vaccination campaigns, as witnessed with COVID-19 vaccine and research into this is an ongoing activity as the world transitions to a COVID-19 endemic state (19–21). To facilitate obtaining real-time insights, the deployment of Quick Assessment of Vaccine Hesitancy (QAVH); a rapid procedure for sampling key, healthcare populations on their attitudes to mass vaccination campaigns that can be used to supplement existing data sources. QAVH comprises obtaining data through rapid, multiple choice question surveys combined with reviewing country reports from the Ministries of Health. The QAVH approach uses free, cloud-based online tools and leverages established administrative resources within each countries’ Ministry of Health (MoH) to generate data profiles on hesitancy from all levels of the healthcare system including vaccine program managers through to frontline care providers. Data collection is facilitated through a combination of online webinars with heads of healthcare departments, and surveys of healthcare managers and primary HCWs.

### 2.1. Webinars

Online webinars with key stakeholders from countries’ MoH and development partners were used to raise the profile and understanding of vaccine hesitancy as well as data collection on vaccine communication and demand creation challenges. For the survey of HCWs in Central and West Asia, two webinars were provided at least a week apart with attendees from participating countries as well as attendees from non-participating countries including Turkmenistan and Pakistan. The first webinar concentrated on setting the reporting requirements for healthcare managers. The second webinar concentrated on collecting reports from healthcare managers on subnational (regional and district) vaccine hesitancy issues. Healthcare managers provided structured presentations with a focus on collecting insights on the factors that drive hesitancy based on reporting requirement set by MoH. The insights provided were used to inform the development of survey questions for healthcare managers and primary HCWs.

### 2.2. Cloud-based surveys

Surveys of healthcare managers are used to collect communication and demand issues as perceived by the representatives of the healthcare system. The healthcare managers can include heads of hospitals, district health departments, or other types of local-level health managers. The survey of primary HCWs aims to collect primary level data on hesitancy issues, including misinformation reported to primary healthcare providers during patient visits, vaccination sessions, or through social media or personal communication with other people.

These surveys were made available online and were targeted to primary HCWs and healthcare managers with a policy of active follow-up pursued to foster engagement through established communication channels with primary HCWs and healthcare managers, as well as personal communication tools including online chats, messengers, and social media. Snowball sampling (22) was the preferred method of survey rollout for the QAVH approach since it is cheap, simple, and requires fewer human resources. The links to the surveys were sent through established communication channels, such as WhatsApp groups for healthcare workers or e-mail distribution, with a request to share with other colleagues.

### 2.3. Survey results summary

As a proof-of implementation for using this QAVH approach within an ongoing public health emergency we collected data from COVID-19 vaccine hesitancy surveys for healthcare managers and primary HCWs submitted between 28 February 2022 and 29 March 2022 from respondents in 4 countries in Central and West Asia (Armenia, Georgia, Tajikistan, and Kyrgyzstan). The survey was provided to 529 potential respondents, 522 (99%) of which agreed to participate and seven (1%) declined participation. Only answers from those who proactively consented to participate included for analysis (65 healthcare managers and 457 primary HCWs).

**Figure 1:**
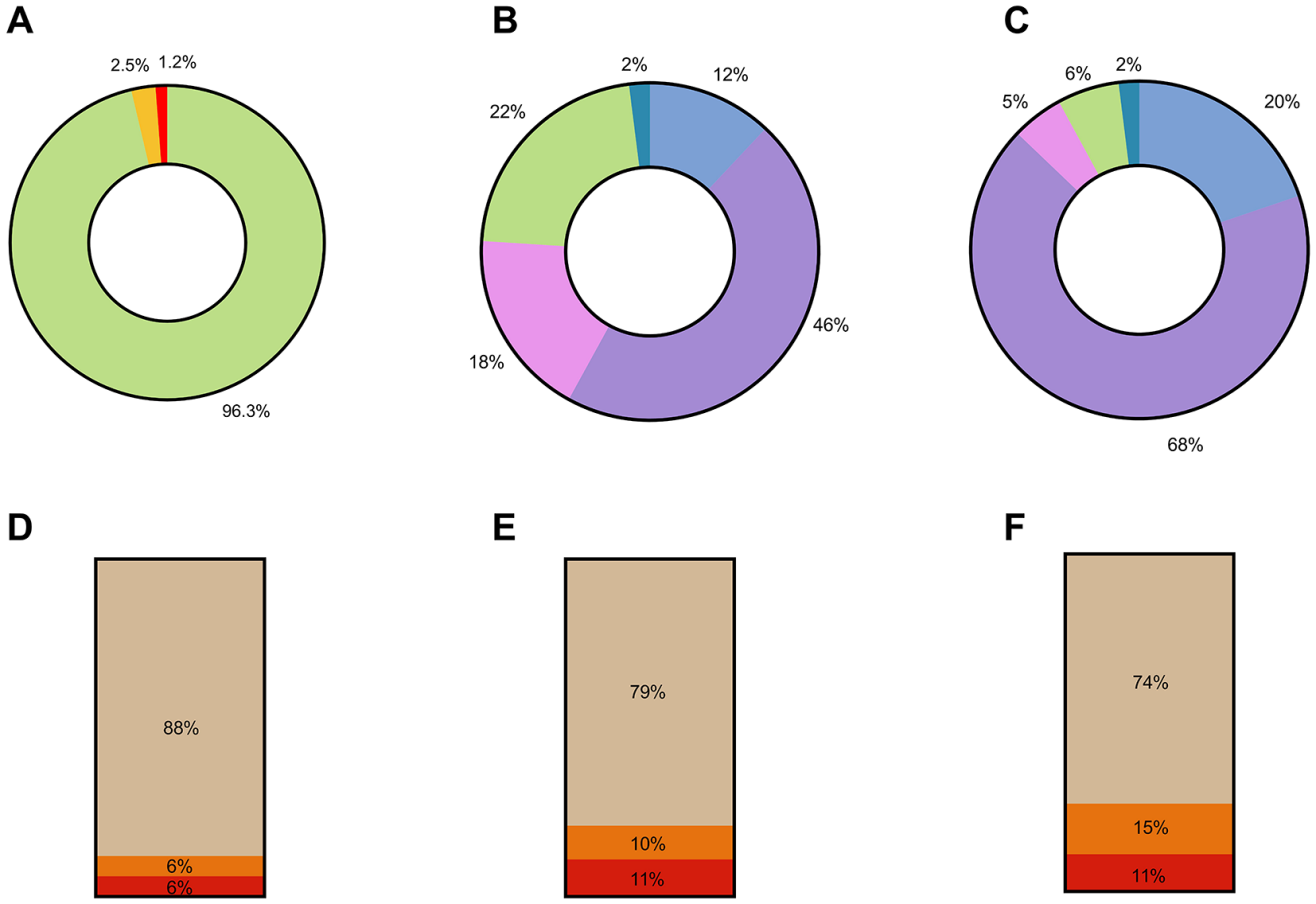
Summary of QAVH survey responses. **(A)** Percentage distribution of vaccination status across all 522 survey respondents. Green—% fully vaccinated, Yellow—% partially vaccinated, Red—% unvaccinated. **(B)** Response by 65 Healthcare Managers to the following statement: “The communication campaign is successful in my country/region.” Blue—strongly agree, purple—agree, pink—undecided, green—disagree, teal—strongly disagree. **(C)** Responses by 65 Healthcare Managers to the following statement: “The communication campaign needs some improvement in my country/region.” Blue—strongly agree, purple—agree, pink—undecided, green—disagree, teal—strongly disagree. **(D)** Responses by 457 Primary Healthcare Workers to the following statement: “Do you trust the efficacy and safety of the COVID-19 vaccines used to vaccinate the public?.” **(E)** Responses by 457 Primary Healthcare Workers to the following statement: “Do you think COVID-19 vaccination should be mandatory?.” **(F)** Responses by 457 Primary Healthcare Workers to the following statement: “Have you been subjected to administrative or other pressure to get vaccinated against COVID-19?.” Beige—yes, orange—no, red—prefer not to respond.

In summary, of the 522 respondents who agreed to participate, 503 (96.3%) were fully vaccinated, comprising 438 HCWs and all 65 healthcare managers, 13 HCWs (2.5%) had received only one vaccine dose, and 6 HCWs (1.2%) were not vaccinated (Figure 1A). The importance of vaccination communication to reach immunization targets was supported across the surveyed countries. Over half of respondents agreed (46% agreed, 12% strongly agreed) with the statement that the communication campaign had been successful in their country, and most respondents (68% agreed, 20% strongly agreed) supported the statement that the communication campaign in their country could be improved (Figure 1B). The notion that the communication campaign was successful in the respondent’s country was disagreed by over 22% of the surveyed healthcare managers, while 18% were undecided on the issue (Figure 1C). While 88% of respondents trust in the safety and efficacy of vaccines was greater than the public trust (Figure 1D) there was concern that there was a degree of vaccine hesitancy among HCWs who are at the frontline of the vaccine effort. However, further survey responses illustrated that trust in the safety and efficacy across all the vaccines available varied significantly, which could indicate that hesitancy may be toward specific vaccine types and not the COVID-19 vaccine in general. While the discussion around mandatory vaccination is multifactorial, with 79% of primary HCWs supporting its implementation (Figure 1E), it does support the consensus that most of the respondents support and advocate for the role of vaccination in a countries’ COVID-19 response. This is further supported by further survey responses illustrating that 74% of respondents were not under administrative or other pressure to be vaccinated against COVID-19 (Figure 1F). The full demographics of respondents are summarized in Table 1.

**Table 1:** Demographics of survey respondents.

	Armenia	Georgia	Kyrgyzstan	Tajikistan	No location
Total participants	52 (0)	12 (34)	0 (7)	1 (399)	0 (17)
Male	21 (0)	4 (7)	0 (0)	0 (345)	0 (2)
Female	30 (0)	8 (27)	0 (7)	1 (54)	0 (15)
Age-range					
18–24	1 (0)	0 (1)	0 (0)	0 (40)	0 (0)
25–34	11 (0)	0 (0)	0 (4)	0 (135)	0 (1)
35–44	20 (0)	3 (4)	0 (2)	0 (89)	0 (1)
45–54	9 (0)	4 (10)	0 (1)	1 (85)	0 (8)
55–64	10 (0)	4 (16)	0 (0)	0 (43)	0 (6)
65–74	1 (0)	1 (3)	0 (0)	0 (7)	0 (1)
Degree level					
Gradate	39 (0)	9 (28)	0 (0)	1 (151)	0 (12)
Postgraduate^*^	13 (0)	3 (6)	0 (7)	0 (214)	0 (5)
No degree	0 (0)	0 (0)	0 (0)	0 (34)	0 (0)

Responses presented from healthcare managers and healthcare workers (in parentheses). ^*^Postgraduate degrees include secondary specialized educations.

These survey results were predominantly sought to further understand the vaccine hesitancy profile in the region. However, we also used these results alongside feedback obtained during the webinars from countries in the region to inform our collaboration with the countries to subsequently develop and disseminate training videos in several languages to counter vaccine hesitancy, prioritizing knowledge gap areas in vaccine manufacturing, vaccine regulation, benefits of immunization as well as ensuring quality during storage and distribution (these videos are available at https://www.youtube.com/@VaccineAdvisoryFirmAsia/playlists and within the Supplementary data). The end-user multifunctionality of these surveys is another advantage that can be incorporated in to developing real-time policy and training programs for key stakeholders.

## 3. Summary

While many countries have prioritized access to vulnerable cohorts based on age and risk, global vaccine supply should now facilitate access for most people eligible for COVID-19 vaccination. However, despite sufficient supply, most countries are now experiencing a significant slowdown in COVID-19 vaccination uptake. While there are different barriers to COVID-19 vaccination in different countries and in subnational regions and districts, a significant contributor to the slowing rates of COVID-19 vaccination currently being observed is vaccine hesitancy.

To improve uptake in the short-term of available COVID-19 vaccines as well as preparing for future emergency mass vaccination campaigns it is essential for different countries’ vaccination program administrators to understand their individual degree of vaccine hesitancy and key concerns in order to develop strategies and policies to address vaccine hesitancy. We have demonstrated that anonymized online surveys are a useful and cost-effective way to gather information on the country’s vaccine hesitancy profile especially when combined with regular webinars to engage with HCWs.

The QAVH approach does not require significant financial or human resources compared with traditional population data collection tools which can make it an easily accessible and readily deployable tool to augment traditional population-based surveys. This QAVH approach may have greater applicability in LMICs which are frequently resource light for developing the larger epidemiological studies necessary to fully appreciate the levels of vaccine hesitancy data in key at-risk populations and could facilitate development of key, evidence-based communication and demand creation activities and strategies in future acute public health emergencies. Most importantly, rapid data collection and analysis facilitated by these tools allowed us in collaboration with countries to identify knowledge gaps and develop training videos to address these gaps. The results from this study support the conclusion from previous studies that combatting vaccine hesitancy within the region of Central and West Asia will require a multipronged approached focusing on enhanced digital engagement to address the concerns of healthcare professionals, improving communication strategies for health service provider and apply solutions based on real-time behavioral insights to reinforce demand (6, 8).

### 3.1. Limitations

We developed the QAVH approach to quickly assess vaccine hesitancy issues in low resource environments and limited timeframes and can be adopted in the early stages of future acute public health emergencies, especially if countries do not have the results of general population KAP surveys available. There are several limitations of QAVH approach to be aware of, which implies it should not be considered a replacement for general population KAP surveys:

- the survey uses proxy population (HCWs and managers);
- sampled population may not be representative of the target population, though this could be mitigated through careful identification of appropriate sampling;
- response biases could affect data collection due to differences in vaccination rates between sample populations and general population;
- data is collected using the snowball sampling, which could hamper the heterogeneity in the sample; and
- insights collected from healthcare or stakeholder representatives might not be accurate due to administrative pressure for better results or a lack of established information collection network.

### 3.2. Key recommendations for deploying a QAVH approach

- Engage with a range of stakeholders and key policy makers to raise understanding on the hesitancy profile and the development of communication partnerships to facilitate wide-reaching messaging.
- Use webinars with key stakeholders and key opinion leaders in order to inform development of more precise surveys for data collection, such as selecting target populations or identifying specific vaccine hesitancy issues.
- Every effort should be undertaken to ensure the anonymization of survey responses.
- Use a specific sampling technique when deploying surveys, such as snowball sampling, sometimes referred as chain-referral sampling, in which existing subjects provide referrals to recruit samples required for a research study. This method allows accessing hard to reach populations, can dramatically increase the sample size from few starting points and allow for insights to be collected from participants without bias undue due to perceived administrative pressure or a lack of established information collection network.
- The timeline for deploying a QAVH approach depends on the capacity and experience of the MoH with surveys and data collection, but generally should not take more than 4–6 weeks.
- Share the results of surveys immediately with a range of stakeholders to facilitate the development of strategies to address identified knowledge gaps.

## Data availability statement

The raw data supporting the conclusions of this article will be made available by the authors, without undue reservation.

## Ethics statement

Ethical review and approval was not required for the study on human participants in accordance with the local legislation and institutional requirements. The patients/participants provided their written informed consent to participate in this study.

## Author contributions

SA conceptualized the study, designed the methodology, analyzed the data, interpreted the results, and drafted the initial manuscript. AB conceptualized the study, visualized the data, and contributed to data analysis and interpretation. AB, DP, HR, JH, SL, GR, and LK reviewed and edited drafts of the manuscript. BC reviewed and edited the manuscript and provided overall supervision. All authors contributed to the article and approved the submitted version.

## Funding

This study was funded by the Asian Development Bank. The findings, interpretations, and conclusions expressed do not necessarily reflect the views of ADB, its Board of Governors, or the governments they represent. Any designation of or reference to a particular territory or geographic area, or use of the term “country” is not intended to make any judgments as to the legal or other status of any territory or area.

## Conflict of interest

SA, AB, JH, DP, HR, SL, GR, and LK were employed by Crown Agents. BC was employed by Asian Development Bank.

## Publisher’s note

All claims expressed in this article are solely those of the authors and do not necessarily represent those of their affiliated organizations, or those of the publisher, the editors and the reviewers. Any product that may be evaluated in this article, or claim that may be made by its manufacturer, is not guaranteed or endorsed by the publisher.
