# Supplementary material for: Understanding COVID-19 vaccine hesitancy in health care professionals in Central and West Asia: lessons for future emergency mass vaccination campaigns
Source: Front Public Health. 2023 Jun 16;11:1196289. doi: 10.3389/fpubh.2023.1196289 (PMC10321768; doi:10.3389/fpubh.2023.1196289)
Supplement: Supplementary file 1 [file Data_Sheet_1.docx]

Supplementary Materials

Hyperlink to Crown Agents Vaccine Advisory Firm YouTube channel for access to webinars around vaccination in Central and West Asia in multiple languages

<https://www.youtube.com/channel/UCi176cRS4UwGzKIRiwmgF7w>
