# Supplementary material for: Understanding COVID-19 vaccine hesitancy in health care professionals in Central and West Asia: lessons for future emergency mass vaccination campaigns
Source: Front Public Health. 2023 Jun 16;11:1196289. doi: 10.3389/fpubh.2023.1196289 (PMC10321768; doi:10.3389/fpubh.2023.1196289)
Supplement: Supplementary file 2 [file Data_Sheet_1.PDF]

VACCINE HESITANCY SURVEY FOR HEALTHCARE WORKERS

Vaccine Advisory Firm. If you have questions about the study, please contact: Larissa.Kokareva@crownagents.co.uk.

* Required

This voluntary survey is part of the efforts of Vaccine Advisory Firm to understand the state of COVID-19 vaccination from the perspective of healthcare managers and primary healthcare workers in the region of Central Asia and Caucasus. If you are healthcare manager or primary healthcare worker on local (district), country, or regional level your participation will greatly aid our research. This survey will take about 10-25 minutes. The purpose of this voluntary survey is to gain a better understanding of the vaccine hesitancy causes in the region. This survey is anonymous, and your responses will be used to support COVID-19 vaccination efforts in the region. The results of the survey will be aggregated and will not use individual responses. If you have questions about the study, please contact: Larissa.Kokareva@crownagents.co.uk.

1. By proceeding further, you confirm that understand the above and consent to participate in this survey run by the Vaccine Advisory Firm.

Mark only one oval.

- ◯ I agree *Skip to question 2*
- ◯ I disagree *Skip to section 5 (Thank you for the time you took to complete the survey!)*

What is your position?

2. *Mark only one oval.*

- ◯ Healthcare manager *Skip to question 3*
- ◯ Primary healthcare worker *Skip to question 13*

Healthcare managers vaccine hesitancy survey

3. 1. What is your sex? *

Mark only one oval.

- Male
- Female
- Prefer not to say

4. 2. How old are you? *

Mark only one oval.

- 18-24
- 25-34
- 35-44
- 45-54
- 55-64
- 65-74
- 75 years or older

5. 3. What is the highest level of education you have completed? *

Mark only one oval.

- No school education
- Elementary school
- Incomplete secondary education (classes 8-9)
- Complete secondary education (classes 10-11)
- Secondary specialized education
- Incomplete higher (Incomplete graduate)
- Higher (Graduate)
- Postgraduate

6. 4. What healthcare level do you work in? *

Mark only one oval.

- Local
- Regional
- National
- Multi-national

7. 5. Which organization are you (mainly) representing/ working for? *

Mark only one oval.

- Ministry of Healthcare
- Sanitary Epidemiologic Service
- Regional Department of Health
- District or Regional Hospital
- Vaccination center
- Non-Governmental Organization
- Quasi-Governmental Organization
- Other: ______________________

8. 6. What is your country of residence? *

Mark only one oval.

- Armenia
- Azerbaijan
- Georgia
- Kazakhstan
- Kyrgyzstan
- Tajikistan
- Turkmenistan
- Uzbekistan

9. 7. What is your COVID-19 vaccination status? *

Mark only one oval.

- I am not vaccinated and do not plan to vaccinate
- I am not vaccinated because I am not eligible
- I am not vaccinated but will vaccinate soon
- I am not vaccinated because I already have antibodies after being sick with COVID-19
- I have received 1 dose of vaccine
- I have received 2 doses of vaccine
- I have received booster dose of vaccine

10. 8. How strongly do you agree/disagree with the following statements on COVID-19 vaccine communication? *

Mark only one oval per row.

	Strongly disagree	Disagree	Undecided	Agree	Strongly agree
**Vaccination communication is important to reach country (regional) immunization goals)**	⬭	⬭	⬭	⬭	⬭
**The communication campaign is successful in my country (region)**	⬭	⬭	⬭	⬭	⬭
**The communication campaign needs some improvement in my country (region)**	⬭	⬭	⬭	⬭	⬭
**I feel comfortable with my vaccine communication skills**	⬭	⬭	⬭	⬭	⬭
**My colleagues are highly proficient in vaccination communication**	⬭	⬭	⬭	⬭	⬭

FOR THE NEXT SET OF QUESTIONS, THINK OF THE PEOPLE WHO DO NOT WANT TO RECEIVE A COVID-19 VACCINE IN YOUR COUNTRY (REGION).

11. 9. How strongly do you agree/disagree that the factors below affect immunization goals of your country (region)? *

Mark only one oval per row.

	Strongly disagree	Disagree	Undecided	Agree	Strongly agree
**People are concerned about possible side effects of a COVID-19 vaccine**	⬭	⬭	⬭	⬭	⬭
**People are not confident that COVID-19 vaccine will work**	⬭	⬭	⬭	⬭	⬭
**People do not believe that they need a COVID-19 vaccine**	⬭	⬭	⬭	⬭	⬭
**People do not like vaccines generally**	⬭	⬭	⬭	⬭	⬭
**People wait and see if it is safe and may get it later**	⬭	⬭	⬭	⬭	⬭
**People think that other people need it more right now**	⬭	⬭	⬭	⬭	⬭
**It is against people's religious beliefs**	⬭	⬭	⬭	⬭	⬭
**The social media news about COVID-19 vaccines strongly**	⬭	⬭	⬭	⬭	⬭

**affects people's decision to vaccinate**					
**People do not trust the government**	◯	◯	◯	◯	◯
**People do not trust the pharmaceutical companies**	◯	◯	◯	◯	◯
**People do not have access to COVID-19 vaccines**	◯	◯	◯	◯	◯
**People are skeptical about the safety of a vaccine due to shorter development period**	◯	◯	◯	◯	◯

12. 10. In your opinion, how much do people in your country (region) trust in the following sources of news and information about COVID-19? *

Mark only one oval per row.

	Do not trust	Somewhat trust	Trust
**Local health workers, clinics, and community organizations**	⬭	⬭	⬭
**Local scientists and other health experts**	⬭	⬭	⬭
**Government health authorities or officials**	⬭	⬭	⬭
**Politicians**	⬭	⬭	⬭
**Journalists**	⬭	⬭	⬭
**Friends and family**	⬭	⬭	⬭
**Religious leaders**	⬭	⬭	⬭
**International scientists and other health experts**	⬭	⬭	⬭
**World Health Organization (WHO)**	⬭	⬭	⬭

Primary healthcare workers vaccine hesitancy survey

13. 1. What is your sex? *

Mark only one oval.

- Male
- Female
- Prefer not to say

14. 2. How old are you? *

Mark only one oval.

- 18-24
- 25-34
- 35-44
- 45-54
- 55-64
- 65-74
- 75 years or older

15. 3. Health organization where you work *

Mark only one oval.

- Hospital
- Out-patient clinic
- Other: ______________________

16. 4. Position in the health organization *

Mark only one oval.

- Doctor
- Nurse (or equivalent position)
- Other: ______________________

17. 5. What is the highest level of education you have completed? *

Mark only one oval.

- No school education
- Elementary school
- Incomplete secondary education (classes 8-9)
- Complete secondary education (classes 10-11)
- Secondary specialized education
- Incomplete higher (Incomplete graduate)
- Higher (Graduate)
- Postgraduate

18. 6. What is your country of residence? *

Mark only one oval.

- Armenia
- Azerbaijan
- Georgia
- Kazakhstan
- Kyrgyzstan
- Tajikistan
- Turkmenistan
- Uzbekistan

19. 7. Have you had a coronavirus infection? *

Mark only one oval.

- Yes
- No (skip to question 11)

20. 8. How were you diagnosed with the coronavirus infection?

Mark only one oval.

- Based on a positive PCR test
- Based on clinical or epidemiological data (community-acquired pneumonia based on CT or X-ray)

21. 9. What is the date you were diagnosed with coronavirus infection? (mm/dd/yy)

Example: January 7, 2019

22. 10. What version of the clinical course of coronavirus infection did you have?

Mark only one oval.

- Mild course in the form of an acute respiratory viral infection
- Pneumonia without respiratory distress (moderate)
- Pneumonia with acute respiratory failure and / or severe or extremely severe course (acute respiratory distress syndrome, sepsis)

23. 11. Have you received a COVID-19 vaccine? *

Mark only one oval.

- Yes
- No (skip to question 18)
- Prefer not to say (skip to question 18)

24. 12. If yes, which COVID-19 vaccine did you receive?

Mark only one oval.

- Pfizer
- AstraZeneca
- Moderna
- Sinovac
- Sputnik
- Covivac
- Other: ____________________

25. 13. How many doses of COVID-19 vaccine have you received by the time of the survey?

Mark only one oval.

- One dose
- Two doses

26. 14. When did you receive your first dose of vaccine? (mm/dd/yy)

____

Example: January 7, 2019

27. 15. When did you receive your second dose of vaccine? (mm/dd/yy)

____

Example: January 7, 2019

28. 16. Did you receive a third vaccine dose/ booster dose of COVID-19 vaccine?

Mark only one oval.

- Yes
- No (proceed to question 17)

29. 17. Do you plan to receive a third vaccine dose/ booster dose of COVID-19 vaccine?

Mark only one oval.

- Yes
- No

30. 18. Do you plan to get vaccinated against COVID-19? *

Mark only one oval.

- Yes (skip to question 19)
- No (skip to question 21)
- I do not know (skip to question 22)

31. 19. If yes, what vaccine against COVID-19 do you plan to get vaccinated with?

Mark only one oval.

- Pfizer
- AstraZeneca
- Moderna
- Sinovac
- Sputnik
- Covivac
- Other: ____________________

32. 20. What is your reason for choosing this COVID-19 vaccine? (please give a short answer)

33. 21. If no, what is your reason for not getting vaccinated against COVID-19? (please give a short answer)

34. 22. Have you been subjected to administrative or other pressure to get vaccinated against COVID-19? *

Mark only one oval.

- Yes
- No
- Prefer not to say

35. 23. What are your main sources of information from which you receive reliable, in your opinion, information about the quality and safety of vaccines against COVID-19? (multiple answers possible) *

Check all that apply.

- [ ] Information from official sources (Ministry of Health, Coronavirus Emergency Response Center)
- [ ] Information from the media (TV, radio, newspapers)
- [ ] Information from social networks
- [ ] Information from colleagues from the country and abroad
- [ ] Information from peer-reviewed medical journals
- [ ] Other: ____________________

36. 24. Do you trust the efficacy and safety of the COVID-19 vaccines used to vaccinate the public? *

Mark only one oval.

- Yes
- No
- Prefer not to say

37. 25. Which COVID-19 vaccine do you consider effective and safe? (multiple answers possible) *

Check all that apply.

- [ ] Pfizer
- [ ] AstraZeneca
- [ ] Moderna
- [ ] Sinovac
- [ ] Sputnik
- [ ] Covivac
- [ ] None of them
- [ ] Other: ____________________

38. 26. Do you think COVID-19 vaccination should be mandatory? *

Mark only one oval.

- Yes (proceed to question 27)
- No (skip to question 28)
- Prefer not to say

39. 27. If yes, please indicate in which population groups it should be carried out (multiple answers possible)

Check all that apply.

- [ ] Children from 12 to 18
- [ ] 18-24
- [ ] 25-34
- [ ] 35-44
- [ ] 45-54
- [ ] 55-64
- [ ] 65-74
- [ ] 75 years or older

40. 28. If vaccination against COVID-19 should not be mandatory, briefly describe the reason (please give a short answer)

41. 29. Do you think that the population of the country has unhindered access to vaccination against COVID-19? *

Mark only one oval.

- ( ) Yes
- ( ) No
- ( ) Prefer not to say

42. 30. In your opinion, do the temporary COVID-19 vaccination sites available in the country meet the requirements for high-quality and safe vaccination? *

Mark only one oval.

- Yes
- No (proceed to question 31)
- Prefer not to say

43. 31. If no, please briefly describe what criteria are not met? (please give a short answer)

____

____

____

____

____

44. 32. Which version of the emergence of a new coronavirus infection do you adhere to? *

Mark only one oval.

- The virus originated naturally, without human intervention
- The virus was artificially created
- Find it difficult to answer

Thank you for the time you took to complete the survey!

This content is neither created nor endorsed by Google.

Google Forms
